# Pressure‐Controlled Modifications in Electronic Structure, Mechanical, Thermodynamic, and Optoelectronic Attributes of Perovskite NaPaO_3_ for Energy‐Efficient Applications

**DOI:** 10.1002/open.70210

**Published:** 2026-04-23

**Authors:** Hudabia Murtaza, Mohamed A. Habib, Quratul Ain, Abhinav Kumar, Junaid Munir, Ahmed B. M. Ibrahim, Ankit Dilipkumar Oza, Mokhtar S. S. Al‐Salimi

**Affiliations:** ^1^ Department of Physics University of Management and Technology Lahore Pakistan; ^2^ Department of Chemistry College of Science Imam Mohammad Ibn Saud Islamic University (IMSIU) Riyadh Saudi Arabia; ^3^ Department of Technical Sciences Western Caspian University Baku Azerbaijan; ^4^ Department of Physics Riphah International University Lahore Pakistan; ^5^ Department of Mathematical Sciences Saveetha School of Engineering, SIMATS Chennai Tamilnadu India; ^6^ Department of Chemistry Yafae University College Lahej University Al‐Hawtah Yemen

**Keywords:** DFT, elastic traits, optoelectronics, perovskite oxide, pressure application

## Abstract

Bandgap engineering is the practice of changing a material's electrical structure to increase its bandgap for certain applications. In this paper, we have examined how the physical properties of NaPaO_3_ are affected by bandgap engineering via pressure application. We have assessed the physical attributes of NaPaO_3_ at pressures from 0 to 24 GPa adding 6 GPa to each calculation. To properly account for exchange‐correlation impact, the mBJ potential is used. The structural properties are determined at ambient conditions, which reveal that the studied perovskite is stable. The mechanical properties computed from Thomas Charpin's approach exhibit decreasing trend with increasing pressure. The elastic waves, Debye, and melting temperature also report a decreasing behavior with pressure, revealing a decrease in material's stiffness and rigidity. The obtained values of electronic bandgap report a significant reduction in the electronic bandgap as pressure is increased. As pressure is increased from 0 to 24 GPa, the material's electronic bandgap reduces from 3.64 eV (R‐Γ) to 1.52 eV (M–M). The optical analysis reveals shifting of optical properties from UV region to visible region. This strategy creates new opportunities for technological applications because the reduced bandgap of NaPaO_3_ makes it a desirable candidate for energy storage devices and next‐generation optoelectronic devices.

## Graphical abstract

First principle calculations are carried out first time to study the pressure‐dependent structural, thermodynamic, elastic, electronic, and optical properties of NaPaO_3_ perovskite oxide. Modified Becke‐Johnson (mBJ) exchange‐correlation potential provides very accurate results. The bandgap profiles and related properties are calculated in the present work. The entire work gives useful results of fundamental importance, which can be utilized for the fabrication of optoelectronic and renewable energy applications.

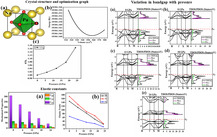

## 1 Introduction

In the current scenario, traditional energy supplies seem to be rapidly depleting [1, 2]. Investigating and locating alternative energy sources are essential because of the growing demand for sustainable energy and the desire to reduce environmental effect [3]. With the sudden increase in population and industrialization, it is also observed that the energy consumption from fossil fuels has increased immensely [4]. There are many energy sources that operate on both conventional and modern techniques, which are utilized widely [5, 6, 7]. The biggest contributor to climate change and global warming is CO_2_, which is released in excess when fossil fuels are burned [8]. Emergent materials for green energy devices have been the subject of study for some decades [9]. One of the fundamental requirements for such devices is the conversion of light and heat energy into electrical energy [10]. The selection of materials is primarily influenced by their cost, safety, and efficiency [11]. Solar energy is widely recognized as a clean, renewable, and environmental friendly power source [12]. Now, we have advanced computational computers so it is now feasible to compute and examine material performance that previously was difficult because of these sophisticated experimental settings and quantum techniques [13]. In recent years, perovskite materials have advanced significantly due to their prominent position in scientific research [14, 15]. Perovskite materials have been effectively used for designing capacitors, sensors, reactors, and storage devices [16]. Similarly, perovskite solar cells have totally revolutionized solar technique [17, 18, 19]. Perovskite oxides (ABO_3_) are extremely adaptable substances with a wide range of physical characteristics. Perovskite oxides can hold a wide variety of cations of various sizes offering higher flexibility than other materials [20]. Multiple perovskite oxides have been discussed prior to this research; for example, the structural parameters of BaCfO_3_ obtained theoretically report complete alignment with the experimental results [21]. Half‐metallic nature is observed for SrPuO_3_ when GGA + U approximation is employed [22]. BaRuO_3_ is determined brittle whereas BaOsO_3_ is considered ductile as reported by their mechanical traits [23]. The CASTEP is used to analyze the physical features of AMnO_3_ (A = Ba, Ca, Sr) which reveals that the studied oxides are ductile and possess higher hardness [24]. High UV absorption is reported for Ba_2_ZrTiO_6_ and Ba_2_ZrCeO_6_ indicating their potential use in UV‐based optical sensors [25]. DFT‐based study on the physical attributes of AcZO_3_ (Z = Al, Ga) reports high absorption in the UV range, suitable for the use of transparent solar cells [26]. Indirect bandgaps with half metallic nature is noticed for A_2_HfNiO_6_ (A = Sr, Ca, Ba) [27]. Furthermore, the modifications from pressure can immensely impact the materials’ physical attributes; for example, the mechanical traits of La_2_NiMnO_6_ report that with a rise in pressure, the material turns from being ductile to brittle [28]. Pressure‐driven modifications on CaSnO_3_ report an increase in the bandgap as pressure is increased to 100 GPa [29]. Using the Monte Carlo simulations, an increase in rigidity and stiffness is noticed in GdAlO_3_ as pressure is increased [30]. The electronic bandgaps for NaTaO_3_ reveal an increase in the materials bandgap (1.69 to 1.93 eV) as pressure is applied till 60 GPa [31]. Cr^3+^‐doped Cs_2_AgBiCl_6_ highlights its strong potential for practical luminescent applications [32]. The spectroscopic properties of Eu‐doped SrSnO_3_ perovskites reveal their strong potential for advanced lighting technologies [33]. Although a substantial amount of literature has been dedicated to exploring the physical attributes of perovskite materials, the effects of pressure‐induced modifications on both existing and newly developed perovskite oxides remain relatively underexplored. In the case of perovskite oxides, applying external pressure can lead to lattice distortions, phase transitions, and changes in the coordination environment of cations, all of which can drastically modify their physical behavior. This opens up exciting possibilities for tailoring material properties to meet specific application demands, such as enhancing conductivity for energy storage devices, tuning bandgaps for optoelectronic application. Systematic studies on pressure‐induced effects could therefore pave the way for engineering perovskite oxides with customized functionalities, pushing the boundaries of their practical use in advanced technologies. NaPaO_3_ has been studied under high pressure of 60 GPa but have with many deficiencies. The authors have used Generalized Gradient Approximation (GGA) to calculate the band structures. The GGA is well known to underestimate the bandgap, which is not comparable with experimental value. They have also utilized Pseudopotential plane wave method which introduces small inaccuracies since it replaces core electrons with pseudopotentials. They also used only 216 k‐points that give a much coarser sampling, which can lead to less precise results and possible numerical errors [34]. In this work, we have scrutinized the pressure effect on the physical attributes on NaPaO_3_ by using the modified Becke‐Johnson potential, which can provide band gap closer to the experimental data. We have used the Full‐Potential Linearized Augmented Plane Wave (FP‐LAPW) method in our work. FP‐LAPW method generally provides more accurate bandgap values because it is an all‐electron approach that treats the potential without shape approximations. The first principles analysis and the pressure‐driven modifications on the physical attributes of NaPaO_3_ have been explored in this research.

## 2 Computational Approach

In order to examine the electronic characteristics of solid‐state materials, computational techniques have been very helpful in forecasting and illustrating their behavior [35]. The high accuracy and computational efficiency of DFT‐based methods have attracted a lot of interest among these. Among these DFT‐based codes, Wien2K has garnered significant attention. Wien2K code specifically addresses core and valence electrons, resulting in more accuracy than pseudopotential‐based approaches [36]. Furthermore, it is compatible with a wide range of materials, including complicated oxides, semiconductors, and magnetic systems. In this work, we used the simulation program Wien2K to investigate how external pressure affects the physical characteristics of NaPaO_3_. To precisely treat the potential in the muffin‐tin and interstitial zones, the FP‐LAPW scheme was applied. To address the exchange–correlation interactions in NaPaO_3_, the mBJ approximation was adopted. Structural and electronic properties were analyzed under applied hydrostatic pressures (GPa) of 0, 6, 12, 18, and 24. The variation in physical behavior under pressure was modeled using the third‐order isothermal Birch–Murnaghan equation of state, expressed as:



(1)

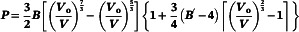




The evaluation of pressure‐induced changes in solid‐state materials is frequently done using this equation. The structural and mechanical properties were analyzed using the PBE‐GGA exchange‐correlation functional. The Thomas Charpin technique [37] was employed to analyze the mechanical traits of NaPaO_3_ at each pressure. The muffin‐tin radii were selected as Na = 2.5, Pa = 2.34, and O = 1.89 (a u^−1^) respectively. The convergence parameters for self‐consistent field computations were selected as RMT × *K*_max_ = 7, *L*_max_ = 11, and *G*_max_ = 14 (a u^−1^). The criteria for charge and energy convergence were set at 0.0001 e/(a.u)^3^ and 0.0001 Ry whereas the cutoff energy for the core and valence electron separation was set at −7 Ryd. A dense k‐point mesh of 2500 points was generated using the Monkhorst–Pack scheme. Finally, the complex dielectric function was utilized to assess the optical response of NaPaO_3_ under varying pressure conditions.

## 3 Research Findings and Discussion

### 3.1 Structural Analysis

**FIGURE 1:**
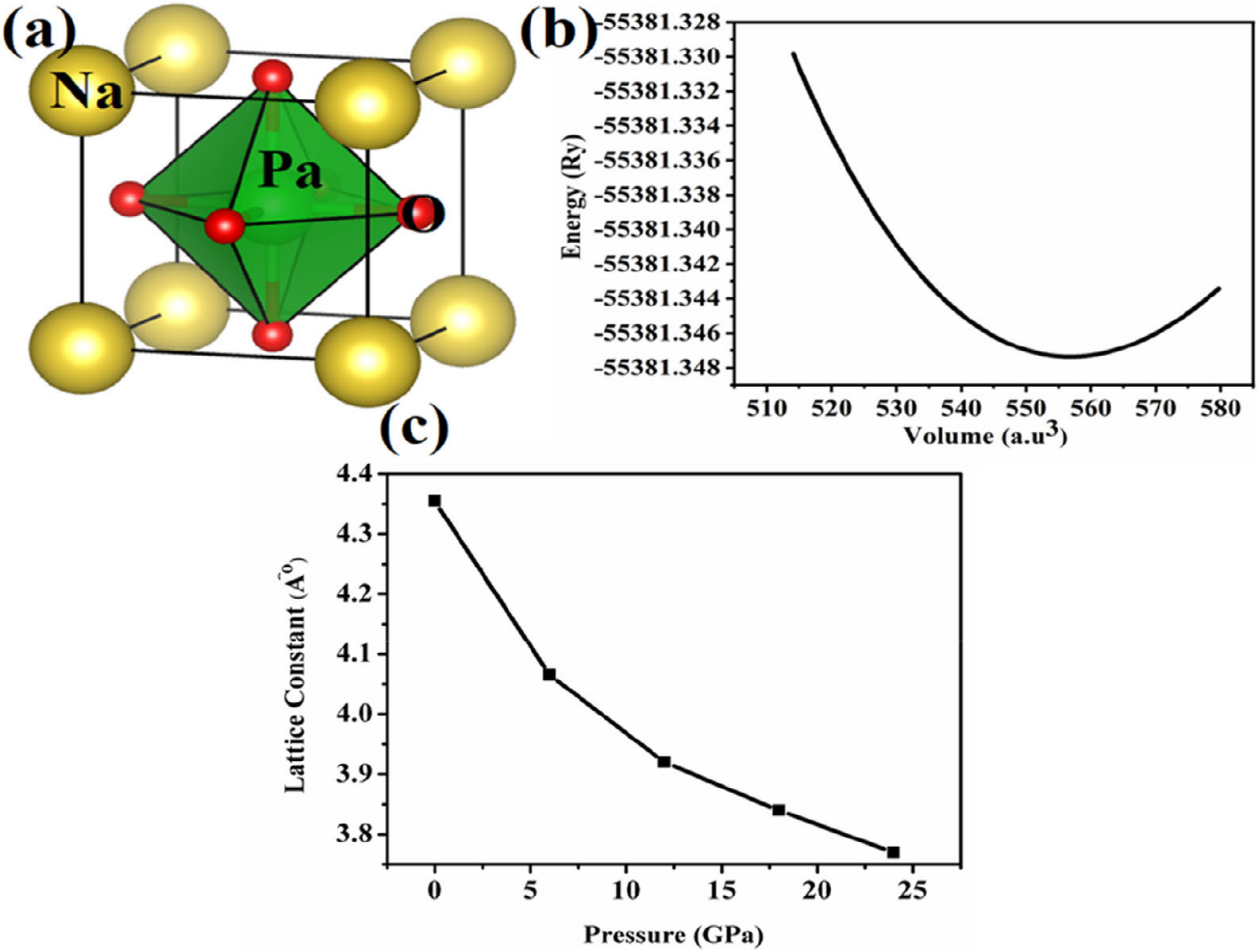
(a) Crystal cell. (b) Optimization curve. (c) Lattice constant of NaPaO_3_ under pressure.



The structural analysis reveals information regarding material's structural anatomy, lattice constant, symmetry, and potential distortions that can cause alternation in the material's physical traits. Therefore, understanding and accurately determining structural properties is essential, as even minor deviations can significantly alter the material's overall performance and functionalities [38]. NaPaO_3_ is a perovskite material with “Na” at A‐site, “Pa” at B‐site and “O” is placed at “X‐site”. Our studied perovskite oxide NaPaO_3_ possesses cubic symmetry with space group of Pm3m. For better understanding of the atomic bonding and the Wyckoff's positions, the VESTA software is employed. Figure 1a reports the crystal structure of NaPaO_3_. The Wyckoff's positions for the studied oxide are observed as Na (0, 0, 0), Pa (1/2, 1/2, 1/2), and O (0, 1/2, 1/2) whereas the atomic bonding between Pa‐O is noticed as 2.63383 Å. After analyzing the structural properties, it is crucial to optimize the material which ensures it reaches its most stable configuration with minimum total energy, which forms the foundation for accurate prediction of its physical and chemical properties. The volume optimization technique which calculates the systems total energy for a range of unit cell volumes is used to optimize structures in solid materials [39]. The most stable configuration of the material is indicated by the equilibrium volume which is the location where the total energy is at its minimum. Figure 1b reports the optimization curve for NaPaO_3_ obtained from Birch–Murnaghan equation, mathematically stated as:

(2)

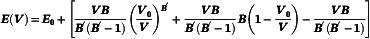




Table 1 reports all the optimized parameters for NaPaO_3_ at ambient pressure. The ground state energy reported as −55 381.347 (Ry/atom). The obtained bulk modulus for the studied oxide reports that materials are highly uncompressible and higher amount of forces are required to compress it. After the studied structure is optimized, further computations can be carried out to assess its physical attributes. The analysis of the formation energy of a material is crucial as it indicates the thermodynamic stability and whether its synthesis is energetically favorable [40]. For negative formation energy, it is revealed that the material is unstable and its synthesis is energetically favorable whereas the positive formation energy suggests that material is stable and its synthesis is energetically not favorable. The formation energy for NaPaO_3_ is obtained as:

**TABLE 1:** Calculated lattice parameters for NaPaO_3_.

Perovskite	NaPaO_3_
Lattice Constant, Å	4.355
B, GPa	146
B’, GPa	5
V_o_, a u^3^	556.81
E_o_, Ry	−55 381.347
$E_{F}$, eV/atom	−2.059

(3) $$E_{F}=E_{\mathrm{NaPa}O_{3}}-(E_{\mathrm{Na}}+E_{\mathrm{Pa}}+3E_{o})$$

Here, the total energy of the material is represented as $E_{\mathrm{NaPa}O_{3}}$ and other terms in the equation depict the individual energies of sodium, protactinium, and oxygen atoms. The obtained formation energy for NaPaO_3_ is negative, which reveals that the studied oxide is stable and its synthesis is energetically favorable corresponding to its discrete atoms. Figure 1c illustrates the variation in the lattice constant of NaPaO_3_ under applied pressure. An overall decreasing trend is observed, indicating an indirect correlation between pressure and lattice constant. Initially, from 0 to 12 GPa, the lattice constant increases gradually. A more noticeable rise begins around 12 GPa, becoming increasingly pronounced after 18 GPa. At 24 GPa, a sharp increase in the lattice constant is observed, suggesting a significant structural response at higher pressures.

### 3.2 Mechanical Attributes

**FIGURE 2:**
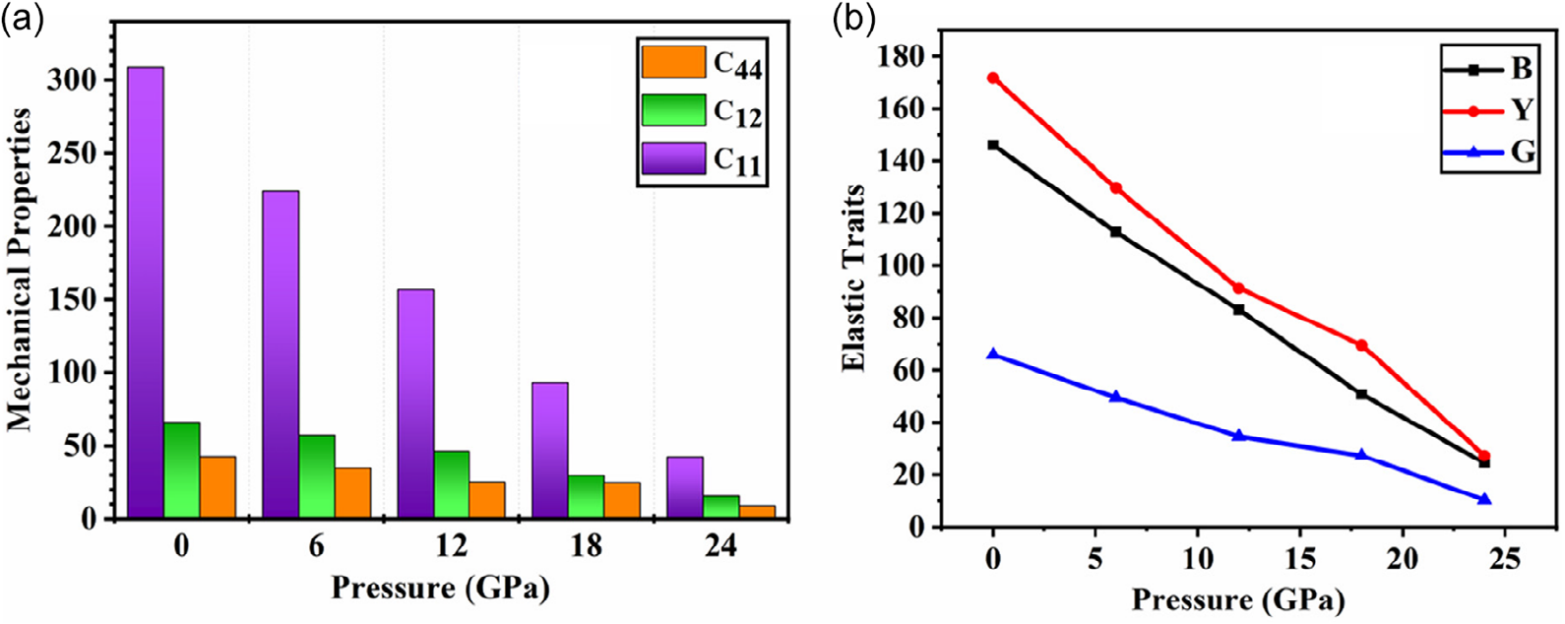
(a) Elastic constants. (b) Mechanical properties of NaPaO_3_ under various pressures.

The mechanical attributes of the material are of immense importance as these mechanical attributes decide whether the material is fit for industrial or real world applications [41]. A detailed understanding of these factors aids in the prediction of material behavior under various loading circumstances, assuring dependability, durability, and safety in real‐world applications such as aerospace, automotive, construction, and energy storage systems. Furthermore, the amount of external pressure or resistive forces can only be analyzed if its mechanical attributes are known [42]. On the other hand, pressure‐driven modifications can drastically change a material's mechanical characteristics by altering its elasticity, hardness, and structural integrity. Such pressure‐induced changes in perovskite oxides can improve their stability and strength, which makes them more appropriate for high‐performance applications. Mechanical properties of the material are driven from the elastic constants of the material which are highly dependent on the material's symmetry [43]. Since the perovskite oxide under study is cubic, its mechanical stability may be determined by using C_11_, C_12_, and C_44_. The pressure range for which the mechanical attributes of NaPaO_3_ is assessed is selected from 0 to 24 GPa with a step size of 6 GPa with each simulation. All the elastic constants and its respective mechanical parameters are presented in Table 2. C_11_ and C_12_ are elastic stiffness constants that describe how a material resists normal deformations whereas C_44_ corresponds to shear deformation, meaning it reflects the material's resistance to shape changes. With pressure increase, it is noticed that all three elastic constants are declining which is also graphically demonstrated in Figure 2a. This decline in elastic constants depicts that as pressure is increased the material's resistance to normal deformations and shape changes declines making it more prone to failure. The bulk modulus is derived by using the elastic constants C_11_ and C_12_ which demonstrates the material's ability to resist compressional strains [44]. The bulk modulus of NaPaO_3_ exhibits a drastic drop as the pressure is raised revealing a significant reduction in the material's resistance toward compressional strains. The Young's modulus reveals the material's endurance level toward the external compressional forces [45]. Higher endurance to external compressional forces signifies higher stiffness and rigidity in the material. With increasing pressure, the Young's modulus of the material demonstrates a declining trend in the value signifying reduction in oxide's stiffness and its strength against the compressional stresses. The shear modulus is a material's resistance to shape changes when exposed to shear stress [46]. As the applied pressure is enhanced, it is noticed that material's resistance to changes in shape decreases suggesting that the studied oxide becomes more susceptible to distortions caused by shear stress. Figure 2b depicts the graphical illustration of the studied oxides bulk, shear and Young's modulus with pressure increased. The anisotropy factor reveals the degree of directional dependence of a material's mechanical properties relative to its crystallographic axes. A value of A = 1 specifies an isotropic material, meaning its mechanical attributes are the same in all directions. Deviations from A = 1 (i.e., A ≠ 1) indicate anisotropy, where the properties vary with direction. Thus, both A > 1 and A < 1 signify anisotropic behavior [47]. The anisotropy factor for NaPaO_3_ exhibits anisotropic nature as the pressure is enhanced till 24 GPa. Comprehending the material's ductile and brittle behavior is extremely crucial as this mechanical trait helps in deciding the material's fitness for specific device. The ductile and brittle nature of the material can be assessed through the Pugh's ratio. A material is considered ductile if the Pugh's ratio is greater than 1.75; otherwise, it is termed brittle [48]. The obtained values of the B/G ratio for NaPaO_3_ exhibit ductile characteristics. The Poisson's ratio can also determine material's brittle or ductile behavior. The criteria is defined as *v* > 0.26 for ductile material and for brittle material the *v* < 0.26 [49]. As the pressure is enhanced form 0 to 24 GPa, the material exhibits ductile characteristics. The Cauchy's pressure is another vital mechanical parameter which describes the bonding nature of the material. For positive values of Cauchy's pressure, the material is termed to have metallic bonding with ductile nature whereas the covalent bonding and brittle characteristics are demonstrated by negative values of the Cauchy's pressure [50]. The computed values of the Cauchy's pressure for all applied pressure demonstrate ductile behavior and suggest metallic bonding among the atoms of the studied oxide. The hardness factor reveals the materials endurance to indentations and scratching [51]. A material that possesses a higher hardness factor depicts that it is tough to cause a scratch in the material. For NaPaO_3_, the hardness factor show a decreasing trend as the pressure is increased revealing that increased pressure can drastically lower the material's endurance. The machinability index indicates how easily a material can be processed or manufactured using machining techniques [52]. The machinability index shows a decreasing trend as pressure is enhanced to 18 GPa whereas at 24 GPa its value slightly increases revealing a fluctuating behavior of machinability index as the pressure is increased. The elastic waves are mechanical disturbances that cause materials to temporarily deform and then return to their original shape. These waves are mainly classified into two types: transverse waves and longitudinal (or compression/pressure) waves. For the studied perovskite oxide NaPaO_3_, the longitudinal and transverse waves report a decreasing trend as the pressure in enhanced revealing a softening of the material's elastic properties. This suggests that the material becomes less rigid and more compressible under higher pressures. Furthermore, the mean sound is also computed to assess the overall stiffness of the material. The evaluated values of the mean sound velocity suggest that increasing pressure causes the reduction in the stiffness of perovskite oxide NaPaO_3_. The Debye temperature also helps in assessing the material's stiffness along with the amount of temperature required to analyze its vibrational modes [53]. The pressure increase leads to a significant reduction in the Debye temperature of the studied oxide revealing that it will require less temperature for the activation of the lattice vibrations if external pressure is involved. Additionally, it is also observed that the studied oxide becomes less rigid as pressure is enhanced. The melting point reveals the level of heat required for melting the material and for the studied oxide it exhibits a declining trend as pressure is increased. This trend also suggests that atomic bonding between the atoms becomes weaker as pressure is increased which results in lower amount of heat required to melt the material. The gradual elastic softening of the NaPaO3 lattice under compression is indicated by the decrease in bulk modulus, shear modulus, and elastic constants with increasing pressure. Such behavior may occur when a material approaches structural instability or a pressure‐induced phase transition, where the lattice's resistance to deformation decreases. Since the average sound velocity, which is dependent on the elastic moduli, is directly correlated with the Debye temperature (θ^D^), a drop in elastic stiffness with pressure results in a fall in sound velocities, which in turn causes a lowering of the Debye temperature. Similar to this, the empirical relation that is used to estimate the melting temperature (*T*_m_) depends on the elastic constants, especially C11. Consequently, a drop in this elastic constant leads to a corresponding decline in the anticipated melting temperature. This phenomenon may be explained as pressure‐induced lattice softening, in which the restoring forces against shear and longitudinal distortions are weakened and the interatomic potential is altered as compression increases. Near structural transitions, such elastic softening has been seen in a number of perovskite‐type systems, resulting in decreases in derived thermodynamic parameters such as Debye temperature and the melting temperature The mechanical characteristics of NaPaO_3_ degrade dramatically as pressure increases, as demonstrated by a decrease in the elastic constants. The material becomes more prone to deformation, with less resistance to compressional and shear loads. Despite having ductile properties, the hardness and machinability index indicate a reduction in the material's endurance at higher pressures. The total stiffness, as measured by the Debye temperature and sound velocities, likewise decreases, implying that the material softens and becomes more compressible. These findings emphasize the necessity of understanding pressure‐induced alterations in perovskite oxides for their prospective use in high‐performance situations.

**TABLE 2:** Elastic properties of NaPaO_3_ under various pressures.

Perovskite	NaPaO_3_				
Pressure, GPa	0	6	12	18	24
C_11_, GPa	308.993	224.121	156.478	93.115	42.456
C_12_, GPa	65.813	57.165	46.40	29.559	15.547
C_44_, GPa	42.522	34.473	25.26	24.751	8.748
B, GPa	146	112.8	83.1	50.73	24.5
Y, GPa	171.7	129.6	91.3	69.57	27.3
G, GPa	65.8	49.5	34.7	27.35	10.39
A	0.34	0.412	0.45	0.77	0.64
B/G	2.23	2.27	2.39	1.85	2.35
v	0.305	0.308	0.32	0.27	0.31
$C_{p}$, GPa	23.29	22.69	21.14	4.80	6.79
$H_{v}$	8.55	6.34	4.61	4.19	1.31
$\mu_{m}$	3.43	3.27	3.28	2.04	2.80
$v_{l}$, m/s	2760.39	2410.42	2049.72	1683.03	1116.38
$v_{t},$ m/s	1461.96	1268.72	1061.64	942.62	581.07
$v_{m},$ m/s	1634.03	1418.65	1188.37	1049.2	650.23
$\theta_{D}$, K	328.48	285.192	238.89	210.915	130.716
$T_{m}\pm$ 300, K	2379.148	1877.55	1477.78	1103.309	803.90

### 3.3 Electronic Traits

**FIGURE 3:**
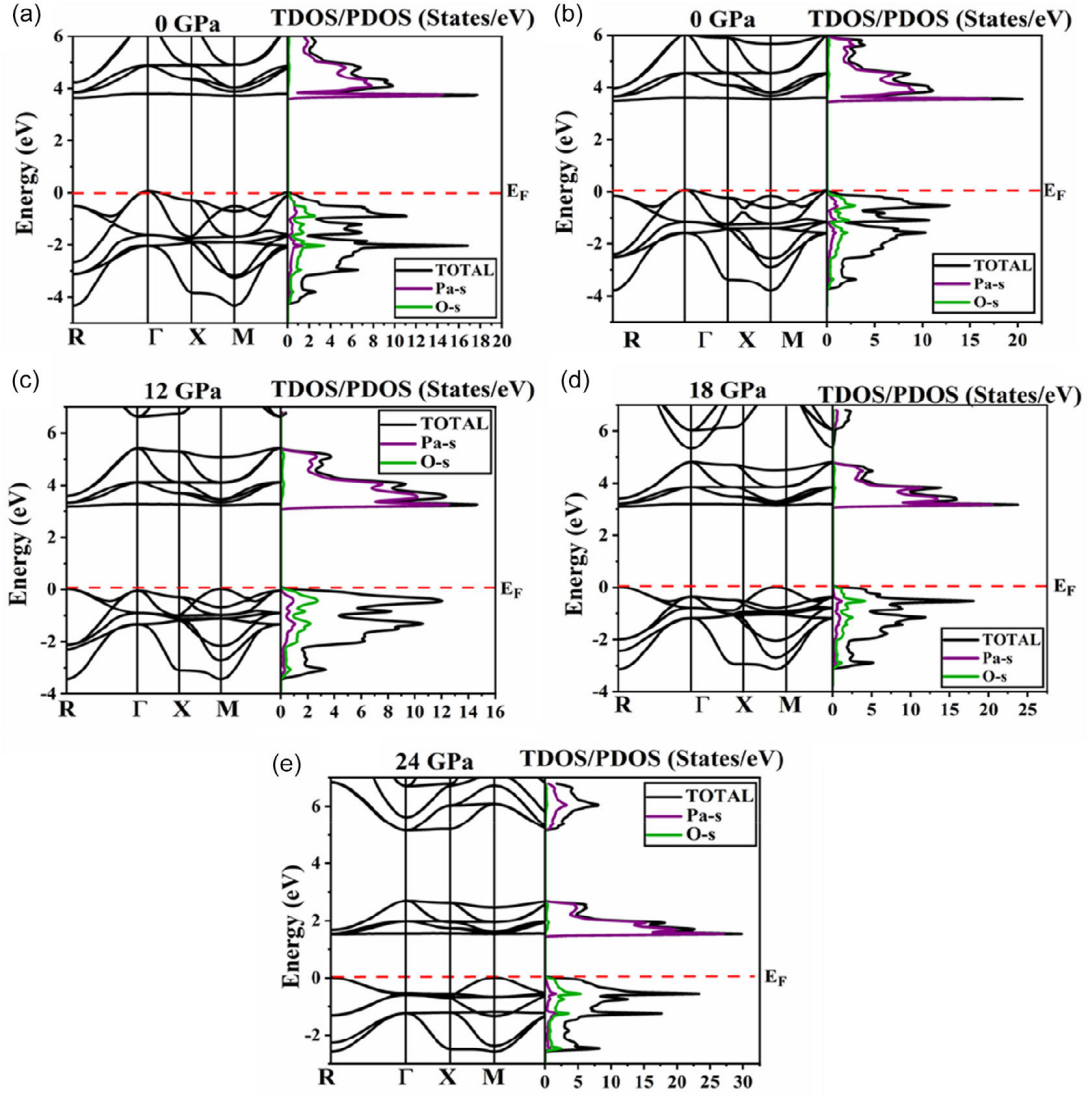
Electronic properties of NaPaO_3_ under various pressures.

Comprehending the materials electronic bandgap is vital for its usage in the design of optoelectronic and photovoltaic devices [54]. Materials with wider bandgaps are generally less suitable for photovoltaic applications; however, applying pressure can effectively reduce their bandgap, making them more viable for such uses. With pressure, the bandgaps of the materials can be tuned which allows enhanced light absorption and increase the efficiency of the overall device. We have computed the pressure‐dependent electronic properties of perovskite oxide NaPaO_3_. The electronic bandgap of perovskite oxide NaPaO_3_ is tuned by applying pressure ranging from 0 to 24 GPa with an increase of 6 GPa in each iteration. Figure 3 demonstrates the pictorial representation of the pressure effect on the electronic bandgap of NaPaO_3_. At ambient pressure, NaPaO_3_ reports an indirect bandgap of 3.64 eV (R‐Γ) as shown in Figure 3a whereas when the pressure is raised to 6 GPa, a slight reduction in the studied oxide's bandgap is noticed. The electronic bandgap reduces to 3.47 eV and remains indirect (R‐Γ) as reported in Figure 3b. At 12 GPa, the electronic bandgap undergoes a transition from indirect to direct, accompanied by a significant reduction in its value. Figure 3c reports a direct bandgap of 3.17 eV (R–R) at 12 GPa. At 18 GPa, the bandgap of NaPaO_3_ reduces to 3.08 eV whereas at 24 GPa, the bandgap reduces to 1.52 eV (M–M) as reported in Figure 3d,e. This trend suggests that increasing pressure not only narrows the electronic bandgap of NaPaO_3_ but also alters its electronic structure, transitioning from an indirect to a direct bandgap and enhancing its potential for optoelectronic devices. The total DOS plots are also plotted against their respective band structures. The total DOS perfectly aligns with the dispersion curves in the band structures at each pressure. Additionally, the influence of individual states has also been assessed. It is noticed that with each pressure, O‐s states reports majority contribution in the valence band whereas Pa‐s states exhibits dominance in the conduction band.

### 3.4 Optoelectronic Analysis

Pressures impact on optical properties can significantly alter material's electrical band structure which in turn influences how light is absorbed and transmitted. The optical absorption of incident light at lower photon energy is improved by bandgap reduction. Furthermore the materials optical properties can be adjusted for use in photonics and optoelectronics due to pressure‐induced changes [55, 56]. These materials are excellent prospects for pressure‐sensitive optical devices and energy‐efficient photonic technologies because of their ability to be precisely tuned under pressure, which allows for fine control over light‐matter interactions. In this section, we have demonstrates the effects of pressure on the optical traits of NaPaO_3_. The pressure range selected for the assessment of the optical properties is from 0 to 24 GPa with an increment of 6 GPa in each iteration. The complex dielectric equation referred as “Kramer's‐Kronig equations” is employed to analyze the effect of pressure on optical traits of the NaPaO_3_ which is stated as:

(4) $$\varepsilon \left(\omega \right)=\varepsilon_{1}\left(\omega \right)+i\varepsilon_{2}\left(\omega \right)$$

The real and imaginary parts are presented as:



(5)

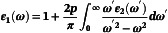






(6)

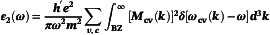




**FIGURE 4:**
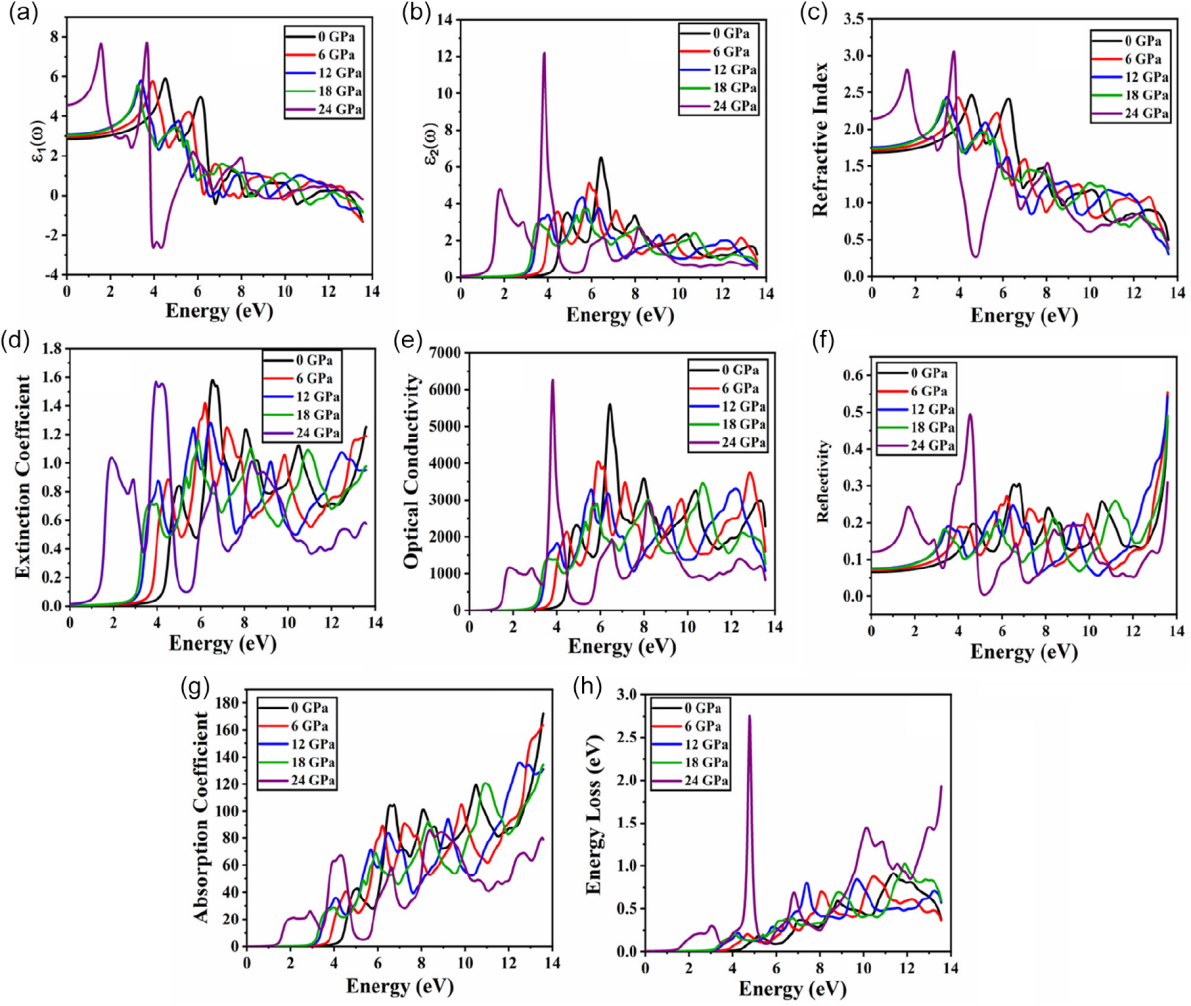
Optical properties of NaPaO_3_ under various pressures.

Here, the real part describes the material's electronic polarizability and the imaginary part describes the material's energy losses and absorption spectra. The effects of pressure on the optical attributes of NaPaO_3_ are presented in Figure 4. The $\varepsilon_{1}(\omega )$ plot for NaPaO_3_ with the effects of pressure is demonstrated in Figure 4a. The static value quantifies the polarizability of the material when it is exposed to constant electric field. The obtained value of real part at zero frequency for NaPaO_3_ is noticed as 2.84, 2.95, 3.03, 3.07, and 4.57 as the pressure (GPa) is increased as 0, 6, 12, 18, and 24. It is observed that as pressure is increased, the value of $\varepsilon_{1}(0)$ also increases, revealing a stronger reaction to an external electric field. Additionally, this increase in the static value directly correlates with the mathematical equation in the Penn's model [57] given as:

(7) $$\varepsilon_{1}\left(0\right)={\left(\frac{h\omega_{p}}{E_{g}}\right)}^{2}+1$$

Here $h\omega_{p}$ is plasma energy and $E_{g}$ is the studied materials electronic bandgap. The maximum peaks for $\varepsilon_{1}(\omega )$ at 0, 6, 12, and 18 GPa are noticed as 4.55, 3.87, 3.38, 3.25 eV whereas for 24 GPa, a minor peak is noticed at 1.57 eV and the maximum peak is reported at 3.68 eV. With an increase in pressure, a significant shift in the real part of NaPaO_3_ is noticed from UV region to the visible region. Figure 4b reports the $\varepsilon_{2}(\omega )$ plot dielectric function for NaPaO_3_ at various pressures. It is noticed that the highest peak of $\varepsilon_{2}(\omega )$ at 0, 6, 12, and 18 GPa is observed as 6.40, 5.83, 5.56, 5.70 eV whereas for 24 GPa, a minor peak is seen at 1.83 eV and the highest peak is noticed at 3.82 eV. With increasing pressure, the primary absorption peak redshifts from 6.40 eV (UV‐region) to 1.83 eV (visible area). Figure 4c reveals that the refractive index of a material is related to how much it slows down light compared to vacuum and is directly linked to the dielectric function of the material [58]. The static value of the refractive index at 0, 6, 12, 18, and 24 GPa is noticed as 1.68, 1.71, 1.74, 1.75, and 2.13. This increase in the static value of refractive index with increasing pressure signifies enhanced light–matter interactions and higher optical density. The maximum peaks of the refractive index for NaPaO_3_ is noticed as 4.53, 4.01, 3.41, and 3.27 eV when pressure is increased as 0, 6, 12, and 18 GPa whereas for 24 GPa, a minor peak is seen at 1.61 eV and the maximum peak is reported at 3.71 eV. This trend depicts that the studied material becomes more optically responsive in the visible region. The amount of attenuation or absorption that light undergoes while passing through a substance is indicated by the extinction coefficient. It determines how strongly the material absorbs photons at different energies and is directly related to $\varepsilon_{2}(\omega )$. The extinction coefficient for NaPaO_3_ is plotted in Figure 4d. The maximum attenuation of light at 0, 6, 12, and 18 GPa is noticed at 6.51, 6.19, 5.64, and 5.89 eV whereas for 24 GPa, a minor peak is detected at 1.94 eV and the maximum value is noted at 3.95 eV. With increasing pressure, the maximum attenuation redshifts shifting from 6.51 eV (UV) at 0 GPa to 3.95 eV (visible) at 24 GPa. Furthermore, a decrease in peak energy indicates a decrease in the energy needed for electronic transitions which suggests a narrowing of the bandgap under pressure. The optical conductivity of NaPaO_3_ is represented in Figure 4e. When an electromagnetic field oscillates, material's optical conductivity indicates how well it conducts electric current. The maximum peaks of optical conductivity at 0, 6, 12, and 18 GPa are noticed as 6.64, 5.97, 5.56, and 5.83 eV whereas for 24 GPa, a minor peak is noted at 1.89 eV and the highest value is noticed at 3.82 eV. With increasing pressure till 18 GPa, gradual drop in the optical conductivity is noticed whereas at 24 GPa, the optical conductivity peak shifts from UV region to visible region. Figure 4f reports the reflectivity graphs for NaPaO_3_ at various pressures. The static value of the reflectivity at 0, 6, 12, 18, and 24 GPa is noticed as 0.065, 0.070, 0.073, 0.075, and 0.119. This increase in the static reflectivity with pressure increase depicts that material becomes more optically dense. With applied pressures from 0 to 18 GPa, the maximum reflectivity is observed at 13.56 eV whereas the highest reflectivity at 24 GPa is noticed at 4.50 eV. This reports that as pressure is enhanced to 24 GPa the studied material exhibits high reflectivity in the visible region. The absorption coefficient for NaPaO_3_ at various pressures is displayed in Figure 4g. A material parameter known as the absorption coefficient indicates how much light or other electromagnetic radiation is absorbed per unit distance as it passes through a medium. The absorption coefficient reports fluctuating peaks at all pressures as energy is increased. The absorption coefficient shows that when pressure is increased from 0 to 18 GPa the maximum absorption occurs at 13.56 eV. At 24 GPa, a small absorption peak is observed in the visible region at 2. 89 eV and the maximum peak is observed at 8.42 eV. This pattern implies that a notable change in the absorption coefficient from UV to visible region is observed with increasing pressure. The energy loss spectrum provides details regarding plasmon excitations, inter‐band transitions, and core‐level excitations by displaying the amount of energy that an electron loses in a substance [59]. The maximum energy loss peak for the studied perovskite oxide at 0, 6, 12, 18, and 24 GPa is reported as 11.38, 10.48, 7.36, 11.85, and 4.77 eV. The pressure application leads to a significant shift in the energy loss spectrum from UV region to the visible region. The optical properties of NaPaO_3_ are considerably altered by pressure resulting in redshifts from higher to low energy region, which suggests that pressure reduces the bandgap and facilitates easier electronic transitions in the material. By decreasing the bandgap, increased pressure improves light absorption at lower photon energies. It indicates stronger light‐matter interactions and optical parameters like the extinction coefficient, reflectivity, and refractive index, all raise with pressure. The possible tunability of NaPaO_3_ for optoelectronic and photonic applications is suggested by these pressure‐induced shifts.

## 4 Conclusion

The pressure's impact on the physical characteristics of NaPaO_3_ was investigated in this work. Using FP‐LAPW technique, we evaluated the material's properties at pressures ranging from 0 to 24 GPa in increments of 6 GPa. Using the mBJ, the proper accounting of exchange‐correlation effects is done for NaPaO_3_. The obtained ground state energy at minimum volume, volume optimization curve, and formation energy demonstrates complete structural stability. The material's resistance to external forces significantly decreases as pressure increases up to 24 GPa as indicated by the values of the elastic constants C_11_, C_12_, and C_44_. The material exhibits anisotropic behavior and ductile characteristics as pressure is increased till 24 GPa. Pressure application has resulted in a drastic drop in elastic waves, Debye, and melting temperatures indicating that the materials atomic bonds have weakened and its stiffness has diminished. The electronic properties reports a decline in bandgap as pressure is increased till 24 GPa. The optical characteristics exhibit a shift from higher to lower energy region under increased pressures indicating the potential application of NaPaO_3_ in LEDs energy storage devices and next‐generation optoelectronic devices.

## Conflicts of Interest

The authors declare no conflicts of interest.

## Data Availability

The data that support the findings of this study are available from the corresponding author upon reasonable request.
